# Effect of Flow Rate Modulation on Alginate Emulsification in Multistage Microfluidics

**DOI:** 10.3390/mi14101828

**Published:** 2023-09-26

**Authors:** Yudan Whulanza, Rithwik Chandur Nathani, Klaugusta Adimillenva, Ridho Irwansyah, Retno Wahyu Nurhayati, Muhammad Satrio Utomo, Abdul Halim Abdullah

**Affiliations:** 1 Department of Mechanical Engineering, Faculty of Engineering, Universitas Indonesia, Depok 16424, Indonesia; 2 Research Center for Biomedical Engineering, Faculty of Engineering, Universitas Indonesia, Depok 16424, Indonesia; 3 Department of Chemical Engineering, Faculty of Engineering, Universitas Indonesia, Depok 16424, Indonesia; 4 Stem Cell and Tissue Engineering Research Center, Indonesia Medical Education and Research Institute, Faculty of Medicine, Universitas Indonesia, Jakarta 10430, Indonesia; 5 National Research and Innovation Agency (BRIN), Tangerang Selatan 15314, Indonesia; 6 Biomechanical & Clinical Engineering Research Group, School of Mechanical Engineering, College of Engineering, Universiti Teknologi MARA, Shah Alam 40450, Malaysia

**Keywords:** double emulsification, stem cell encapsulation, alginate, microfluidics chips

## Abstract

The encapsulation of stem cells into alginate microspheres is an important aspect of tissue engineering or bioprinting which ensures cell growth and development. We previously demonstrated the encapsulation of stem cells using the hanging drop method. However, this conventional process takes a relatively long time and only produces a small-volume droplet. Here, an experimental approach for alginate emulsification in multistage microfluidics is reported. By using the microfluidic method, the emulsification of alginate in oil can be manipulated by tuning the flow rate for both phases. Two-step droplet emulsification is conducted in a series of polycarbonate and polydimethylsiloxane microfluidic chips. Multistage emulsification of alginate for stem cell encapsulation has been successfully reported in this study under certain flow rates. Fundamental non-dimensional numbers such as Reynolds and capillary are used to evaluate the effect of flow rate on the emulsification process. Reynolds numbers of around 0.5–2.5 for alginate/water and 0.05–0.2 for oil phases were generated in the current study. The capillary number had a maximum value of 0.018 to ensure the formation of plug flow. By using the multistage emulsification system, the flow rates of each process can be tuned independently, offering a wider range of droplet sizes that can be produced. A final droplet size of 500–1000 µm can be produced using flow rates of 0.1–0.5 mL/h and 0.7–2.4 mL/h for the first stage and second stage, respectively.

## 1. Introduction

Emulsification is a technology used to protect valuable therapeutic molecules [1,2], living cells [3], or even secretory tissues/islets [4,5,6] in sealed capsules that can release these contents at controlled rates under the influences of specific conditions. This technology dampens or even prevents rapid degradation [7], facilitates immuno-isolation [8], and inactivates target materials until the product reaches the intended spatial location [1,2].

For living cells or islets emulsification, the capsule dimensions are critical for maintaining cell survival [9]. Cells require a sustained supply of oxygen and nutrition for metabolism. In addition, the waste metabolites in a microenvironment should be kept minimal to prevent toxicity. This means that cell transplantation would require the cells to be kept in a certain environment with substances that can support their survival. This need derives from the development of stem cell emulsifications in biomaterial-based microcapsules. Such capsules can be as simple as a solid sphere from a single material [10] or double-layered beads [7], or a more complex structure composed not only of multilayered materials but also of cell-sheet coatings [5]. Microemulsification brings further benefits such as increased immobilization efficacy because of the large surface area and enhanced preservation against heat treatment because of the decreased thermal conductivity with volume [11]. Furthermore, the emulsification coating provides a micro-environment for living cells, which can be loaded with other components including nutrients or growth factors to realize symbiotic particles [12,13].

Spray-drying [14], extrusion [15], lyophilization [16], and microfluidic emulsion are techniques used to make these microcapsules encapsulate nutrients, protein, or living substances such as bacteria and cells. However, several challenges are evident during the scaling-up process, such as gaining high yield and maintaining consistency of the emulsification size [17,18]. The use of droplet-based microfluidics has allowed for the efficient generation of controllable, even-sized droplets. It is even possible to encapsulate a single cell within a single droplet [19]. Hidema et al. [20] studied the effect of channel geometry and fluid chemical properties on double emulsion production by using planar microfluidic devices and reported that the double emulsions produced were highly uniform in size. Kanai and Tsuchiya [21] developed microfluidic devices using the stereolithography method; they reported that their device was able to generate a controllable and monodisperse double emulsion. 

In a double emulsions system, the additional layer would allow more stability of the encapsulated cells at the site. The additional layer is also suggested to produce droplets with better mechanical properties [22]. However, double encapsulation has increased complexity due to the involvement of an additional step in the micro-emulsification process. Double emulsification involves the use of multiple coating materials which can interact with each other and affect the properties of the microcapsules. The coating materials may not be compatible with each other, which can affect the stability of the encapsulated material [23,24]. Several studies have been carried out to formulate various materials which are generally hydrogel-based to meet the main criteria for biocompatibility, biodegradability, and ease of gelation process [14,25]. Alginate was already commonly used as an encapsulant agent due to its low cost, biocompatibility, and ease of alginate formation [16,26]. Building a double emulsification microfluidic system capable of generating alginate capsules of certain sizes requires an appropriate combination of elements. Reports on multiple-emulsion-droplet systems are still limited. 

Here, an experimental approach to multistage alginate emulsification is reported. Several scenarios were proposed that involved different chip materials and flow rate conditions. The system is investigated using dimensionless Reynolds and capillary numbers to obtain a better understanding of cell emulsification application. 

## 2. Materials and Methods

### 2.1. Experimental Setup

**Figure 1:**
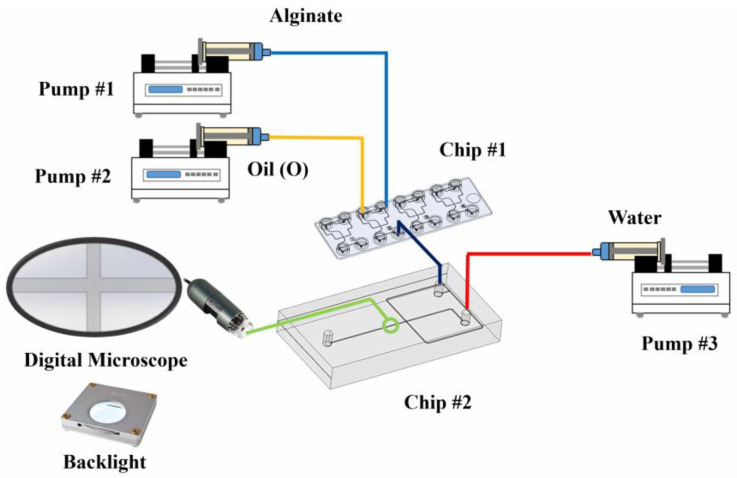
Double stage of emulsification of alginate/oil/water (A/O/W).

The setup of the droplet generation system involves two stages of emulsification. The first stage includes the alginate in a water solution as the dispersed phase and oil as the continuous medium (A/O). The result from the first stage was directly followed by the second stage, which encapsulates the A/O in a water solution to become A/O/W. Figure 1 shows the arrangement of the two-stage emulsification in detail. The result from the second stage is readily contained in the flask to allow for further observation of the resulting emulsification.

### 2.2. Device Design and Fabrication

The first stage of emulsification was conducted in a commercial polycarbonate chip, serial 537, from Microfluidic-ChipShop GmbH (Jena, Germany). The chip is a hydrophobic-droplet generator chip with single cross geometry and luer fitting interfaces. The chip contains four identical droplet generation units with a nozzle size of 38 µm. The oil as the continuous phase was inserted through two inlets that were driven in parallel from one syringe pump, whereas the alginate solution as the dispersed phase entered through one inlet as shown in Figure 1. All tubes have an inside diameter of 0.76 mm and are connected to the luer fitting from Microfluidic Chipshop which then connects with a syringe pump from Terumo (Tokyo, Japan) Terufusion TE 3 Series. The chip has only one outlet which thereby produces an alginate solution droplet in the oil medium (A/O).

The second stage of emulsification was conducted on a PDMS chip with custom design. The microchannel was designed in CAD software (SolidWorks 2020, Dassault Systèmes, Woodland Hills, CA, USA). The channel was formed as a square shaped channel with the dimensions of 300 µm × 300 µm and has a simple flow-focusing pattern. The mould was realized in a steel mould using a CNC machine, similar to the previous fabrication method [27]. A thorough cleansing of the mould from any impurities during the machining process was conducted to prepare the mould to be casted with polydimethylsiloxane (PDMS) Sylgard™ 184 Silicone Elastomer from Dow Corning (Midland, MI, USA). Previously, the PDMS was thoroughly mixed with its crosslinker, then subsequently degassed to remove any air bubbles for 1 h. The PDMS solution was then poured into the mould and heated at 80 °C for around 30 min. The PDMS chip was carefully lifted from the mould, ready for the assembling step [28].

Plasma oxidation bonding of PDMS is a way to change the surface chemistry of a PDMS chip. The oxidation produces silanol terminations (SiOH) on the surface and makes the material hydrophilic for a short amount of time. A single chip is usually composed of two layers and oxidation using plasma serves as a surface treatment that allows the two layers to bond covalently [29].

### 2.3. Microchannel Surface Treatment

Polyvinyl alcohol (PVA) is a highly hydrophilic polymer; in its hydrolyzed state, it is commonly used as a coating in silica capillaries. PVA can be adsorbed into PDMS via heat immobilization post plasma treatment. The adsorption of polyvinyl alcohol onto the surface would turn the PDMS permanently hydrophilic, which would make it suitable for generating oil-in-water droplets. The method includes dissolving PVA in water and mixing it. The solution is then poured onto the PDMS chip and let to sit at room temperature. The chip is then be cleaned of any liquid and heated. After the process is completed once, it is repeated a few more times until the desired level of hydrophilicity is achieved.

Polyvinyl alcohol is added to distilled water (1 wt%) and stirred at room temperature for 40 min. The temperature is then increased to 100 °C and stirred for another 40 min. The temperature is then be reduced to 65 °C and left to stir overnight. The solution is then weighted again, and the amount of water is adjusted to compensate for water loss due to evaporation. The solution is then used according to the following procedure to treat the channel surface. The channel is cleaned and dried, PVA solution is then poured into the channel for 10 min at room temperature and blown dry and heated to 110 °C for 15 min. This step should be repeated at least 3 times for optimum results [30].

### 2.4. Solution Preparation

Originally, the experiment would have alginates from Sigma Aldrich (W201502) of various concentrations between 0 to 5%. However, any concentration above 2% proved detrimental to the droplet’s stability and some trials showed no formation of droplets. To prepare the alginate in water solutions of 1% and 2% *w*/*w*, two steps were conducted. Firstly, a mixture of 0.5–1.0 g alginate with 50 mL of distilled water was prepared in a beaker glass. Secondly, stirring with a magnetic bar was performed for 1 day at room temperature. Then, the alginate solution was ready to use. The solution was stored in a refrigerator at a temperature of around 6–7 °C. The oil phase used was from Sigma Aldrich (St. Louis, MO, USA) (M-5904). Properties for fluids in this experiment are presented in Table 1. 

### 2.5. Parameter Setup

The experiment will be carried out with the setup shown in Figure 1. The result will be converted and presented in terms of the flow Reynolds Number. This is to simplify replication and ensure the scalability of the test.

Dimensionless numbers usually used in study of microfluidic system are the Reynolds number, which represents the ratio of inertial to viscous forces ($Re=\frac{\rho vD}{\mu }$) and the capillary number, which represents the ratio of shearing force to interfacial tension ($Ca=\frac{\mu v}{\gamma }$). The parameters involved in the two dimensionless numbers are fluid density (ρ), velocity of the fluid (v) in the channel, diameter of channel (D), dynamic viscosity of the fluid (μ), and surface tension between two fluid phases (γ). The Reynolds number of water is set around 0.5–2.5, both as the dispersed phase and continuous phase in the case of the first and second stages, respectively. Similarly, the Reynolds number of oil is set at 0.05–0.2. Values for flow rate variation are presented in Table 2.

### 2.6. Data Acquisition and Analysis

The droplet generation was observed using digital microscope Dino-Lite AD7013MTL (Taipei, Taiwan). The digital microscope connected to a personal computer/laptop allowed us to acquire the image or video during the droplet process in the microchannel using DinoCapture 2.0 software. The calibration of the digital microscope was carried out using calibration slide CS-41 from Dino-Lite. The magnification used was 25× magnification. Furthermore, the Fiji Toolbox version of 2.0 (NIH ImageJ) software package is used for image data processing and analysis. 

## 3. Results

### 3.1. First Stage of Emulsification: Water-In-Oil Emulsion 

**Figure 2:**
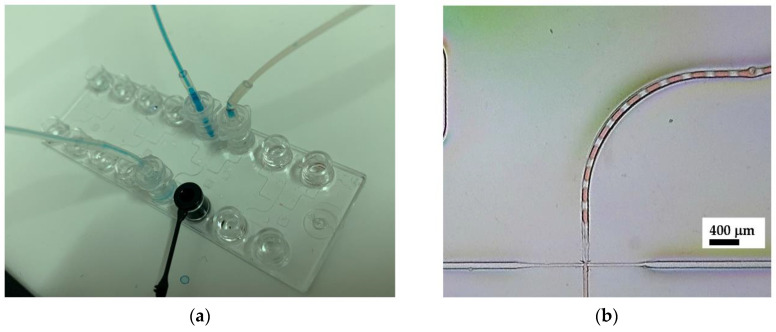
First stage of alginate-in-oil emulsion; (**a**) Commercial drop generator chip and interface setup and (**b**) Microscopic image of alginate-in-oil single emulsion.

Commercial microfluidic chips are available for various purposes, including chips for multiple emulsion systems such as droplet generators. While polydimethylsiloxane (PDMS) is usually used as a standard material for microfluidic chips in research environments, commercial microfluidic chips tend to use other materials such as polycarbonate (PC), as utilized in this experiment. Sylgard 184 was selected due to its optical transparency, biocompatiblity, and non-cytotoxicity, based on our previous study [31]. This type also offers a faster curing compared to Sylgard 182. Moreover, PDMS is known to have a tunable hydrophobicity that benefited from this oil-water emulsification. Figure 2a shows the droplet generation of water in the oil system. Figure 2b depicts the resultant droplet in the microchannel, indicated by the red color as the plug flow. 

**Figure 3:**
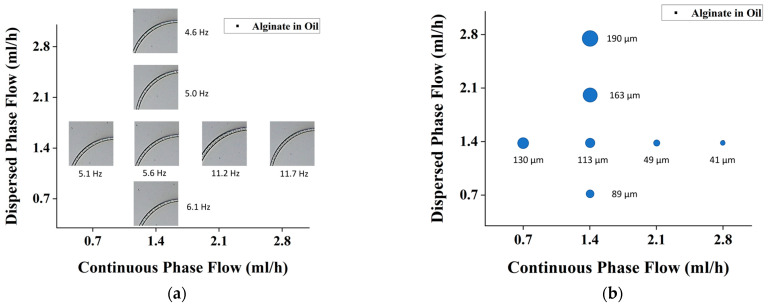
Effect of dispersed vs. continuous flow rate for alginate-in-oil emulsion on (**a**) drop formation frequency, and (**b**) drop size.

Figure 3 also demonstrates the experiment conducted in three different trials to ensure the repeatability of the process and the accuracy of the data obtained. The result in Figure 3 shows droplet size alterations with changes in water flow conditions and oil flow conditions, respectively. Figure 3a indicates the frequency whereas Figure 3b shows the plug length of the generated droplet. To ensure that the readings were standardized, readings were taken 15 min after flow conditions were altered. These suggested that the longer the droplet, the lower the generated frequency.

The trends obtained from the experiments show consistency in the results, which is promising for the system design. It is indicated that the smaller droplet size is compensated for by a higher droplet generation frequency. The polycarbonate chip system has also managed to generate droplets with sizes of d < 200 µm, which is ideal for the first phase of the double-emulsification system.

**Figure 4:**
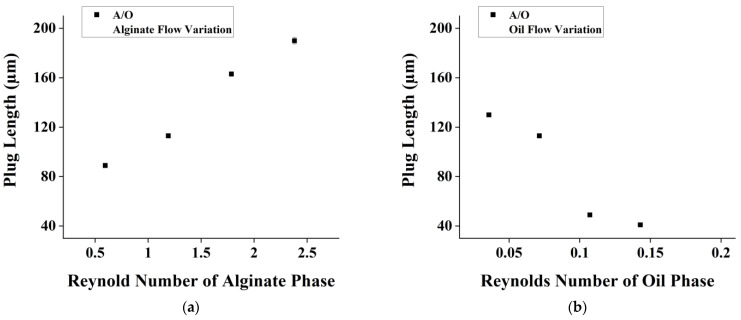
Effect of Re number on plug length for flow rate variation of (**a**) dispersed phase and (**b**) continuous phase in alginate-in-oil droplet formation.

In Figure 4a, the dispersed aqueous phase is flowed with various flow rates while the continuous organic phase remains constant. Meanwhile, in Figure 4b, the continuous organic phase is flowed with various flow rates while the dispersed aqueous phase remains constant. Changes in flow rate ratio cause more linearly proportional changes in plug length. The Reynolds number indicates a creeping flow rate through the channel. This creeping flow suggests that droplet generation can be precisely controlled, making it an ideal regime for various applications. The droplet generation remains uniform, with relatively small deviations at each flow rate. Additionally, the capillary number was also calculated and yielded a value of less than 1. This value indicated that the realized droplet was under a squeezing regime [32,33]. 

### 3.2. Alginate Emulsification

**Figure 5:**
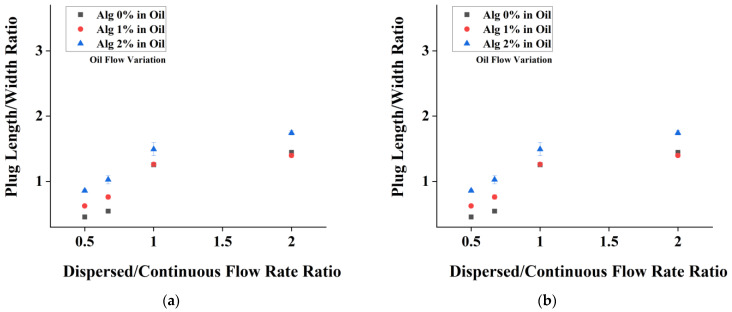
Plot of plug length/width ratio vs. flow rate ratio of (**a**) alginate flow rate variation and (**b**) oil flow rate variation for alginate-in-oil emulsion.

Since the main purpose of the current chip is to perform alginate emulsification, it is essential to study the effect of alginate concentration on droplet production. Figure 5a shows the effect of varying dispersed flowrates, whereas Figure 5b shows the effect of varying continuous phases. The plug length and width ratio achieved were in the range of 0.5–2.5. 

The trends that we retrieved from the experiment are shown in Figure 5. As we can see, the trend of alginate (1 and 2% alginate) is like that of previous water (alginate 0%). The droplet size generated by 1% alginate is not significantly different from that of the water droplet (*p* value < 0.05). On the other hand, 2% alginate demonstrates a significantly different droplet size, specifically during the variation of continuous phase (Figure 5b). Experiments were performed using higher concentrations of alginate; however, no droplets resulted due to clogging of the channel (not shown here). 

The increase in the dispersed phase’s (alginate mixture) Reynolds Number will directly affect the size of the droplet and increase its size. The inverse also applies, where an increase in the oil’s Reynolds Number will decrease the droplet size exponentially. In this test, the droplet sizes are observed to be larger because the dispersed phase’s viscosity is more viscous. This is also supported by a study.

### 3.3. Second Stage of Emulsification: Oil-In-Water Emulsion 

**Figure 6:**
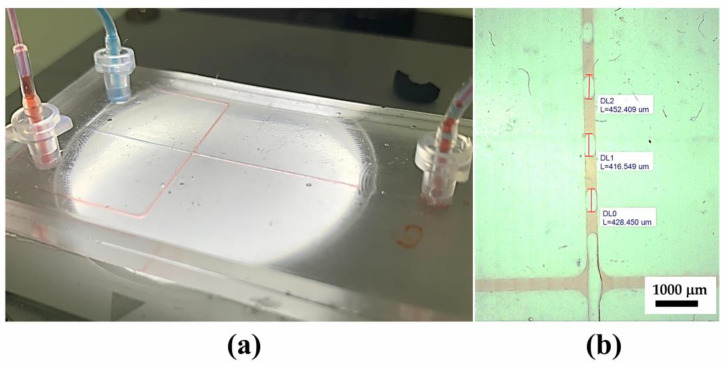
Second stage oil-in-water emulsion: (**a**) customized PDMS chip setup and (**b**) microscopic image of oil-in-water single emulsion.

The second stage of emulsification was carried out using polydimethylsiloxane (PDMS) chips. The chip is designed to have a width of 300 µm to achieve a target droplet size of around 500 µm. The generation of oil-in-water droplets required a slightly different treatment to the chip, where PVA treatment was performed to change the channel affinity from hydrophobic to hydrophilic. Figure 6 shows the proofs of oil-in-water emulsion with around a 400 µm plug length. 

**Figure 7:**
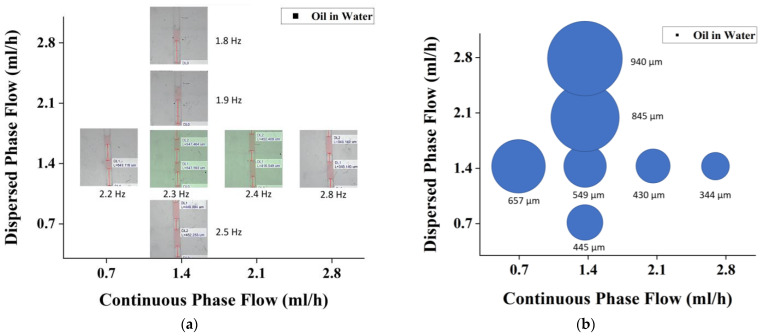
Effect of dispersed vs. continuous flow rate for oil-in-water emulsion on (**a**) drop formation frequency and (**b**) drop size.

In a multiple emulsion system, the aqueous and organic phases might assume the roles of continuous and dispersed phases interchangeably, depending on the requirement. Figure 7 shows how continuous and dispersed phase configurations and flow rate ratios might affect plug length. In Figure 7a, changes in frequency are observed under various dispersed and continuous phases. Meanwhile in Figure 7b, changes in plug length are observed and correlated with the frequency changes. 

**Figure 8:**
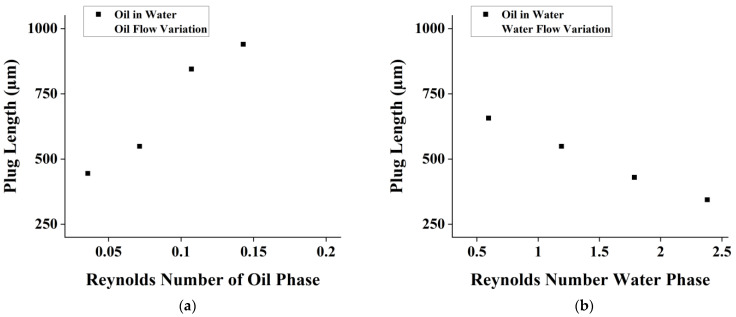
Effect of Re number on plug length for flow rate variation of (**a**) dispersed phase and (**b**) continuous phase in oil-in-water droplet formation.

The oil-in-water experiment again shows that droplets are being generated at plug lengths of 300–900 µm. The droplets are completely generated in the squeezing regimes in which the capillary number equation is satisfied. Uniformity can also be seen and observed by observing the size variation as depicted in Figure 8.

The trends obtained from the oil-in-water PDMS chip-testing experiment is depicted in Figure 8. Note that in this experiment, water is set as the continuous phase and oil is set as the dispersed phase. In this stage, droplet sizes can be observed to be larger in comparison to the water-in-oil testing at the same flow rate. Since water is less viscous than oil, the droplet sizes tend to be larger at the same flow rate for oil-in-water droplet generation.

**Figure 9:**
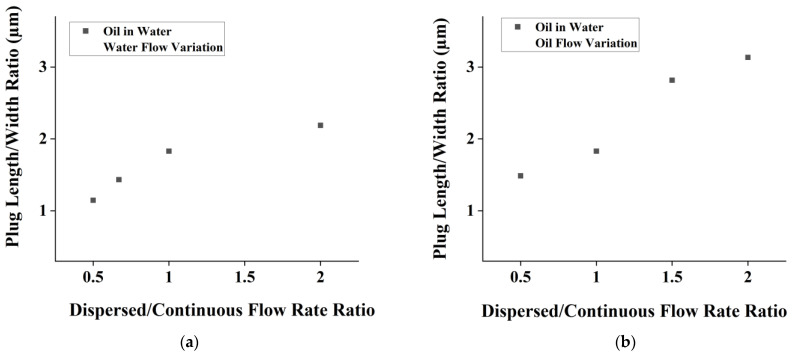
Plot of dispersed/continuous flow rate ratio vs. plug length/channel width ratio for (**a**) oil flow variation and (**b**) water flow variation.

A similar trend was also found in this stage; the resulting plug length/width channel ratio is positively correlated to the dispersed/continuous flow ratio. Note that in this stage, the dispersed variation has a linear trend, as in the previous stage, although the phase is different. This trend is also evident in the continuous variation, as depicted in Figure 9. In Figure 9a,b, a viscoelastic behavior is shown, which indicated by a nonlinear trend.

### 3.4. Double Emulsification

The double-emulsification system is built off the two single emulsification systems that have been tested. The PC chip is used for the first phase of the emulsification as it is hydrophobic, which is suitable for water-in-oil emulsification and has managed to generate droplet sizes that are not larger than the maximum allowable droplet size. The PDMS chip was used as the second phase as the modified version that was treated with PVA has been proven to be hydrophilic which is suitable for oil-in-water emulsion. Testing was performed to prove the viability of the double-emulsification system.

The parameter values used for double emulsification are selected from previous experimental parameters and summarized in Table 3, where the minimum flow rate of a PC chip can be at 0.1 mL/h and the minimum flow rate entering a PDMS chip should be at least 0.7 mL/h for a droplet to be generated. Therefore, the continuous phase flow rate in the first phase should be at least 0.7 mL/h to satisfy this criterion in the second phase. A high water-to-oil flow rate ratio is also needed for the first phase to allow the second phase emulsification to be more efficient. The last phase was set to the highest Reynolds number possible to generate the smallest inner droplet possible. The generated droplets have also been shown to follow the rules of droplet generation. 

**Figure 10:**
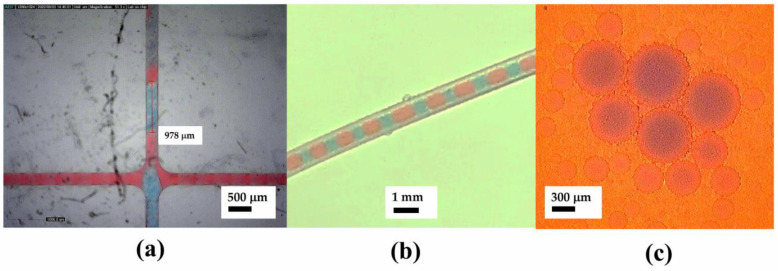
Double emulsification droplet visualization: (**a**) under microscope, (**b**) in the transfer tubing, and (**c**) in bulk solution.

Figure 10a shows the double emulsification plugs, illustrated by blue and white colors indicating 1% alginate core droplets and oil shell, respectively. Meanwhile, the continuous, outer layer of the water phase is indicated by the red color. The detail of emulsification can be observed in the Supplementary Materials. The core and shell layer are evident in Figure 10c, with the form of the droplet becoming a sphere as the droplet exits the microfluidic channel. Figure 10c also shows droplets in the bulk solution, where some are coalesced, forming larger droplets. 

## 4. Discussion

The setup in current study is intended to encapsulate cells in alginate droplets for bioink in bioprinting technology. To ensure successive production of the alginate droplets, the effect of flow rate on droplet formation is investigated. A similar study on the production of sodium alginate microgels using a PDMS-based flow-focusing device also demonstrated the effect of flow rate ratio on the shape and size of the sodium alginate microgels [34].

The mass continuity governing equation was evidently featured in this experiment. In both stages—water-in-oil and oil-in water—the frequency of droplet generation was correlated with its size. The frequency and size parameters were tunable by the flow rate of the aqueous phase. This report shows how variation of the dispersed and continuous phases affects those parameters. We also observed the Reynolds number and the capillary number that were arranged in this experiment and agree with a similar study. The capillary number shows that the regime of the droplets is plug flow. Therefore, we plot the relations of the size of the plug flow with the ratio of disperse/continuous phases. The plot showed a linear behavior in the microfluidics channel. However, we also observed a viscoelastic behavior in the variation of the continuous phase both for the water-and-oil and oil-and-water stages. This is also supported by the study performed by the Sakai research group [35].

**Figure 11:**
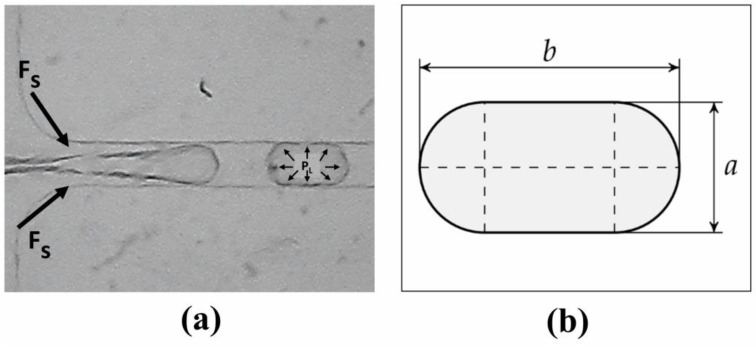
Forces acting during droplet formation: (**a**) microscope view of emulsification, (**b**) schematic view droplet geometry.

Furthermore, the droplet realized showed stability, which can be explained by the Laplace correlation. The stability of droplet formation in the current setup is validated by analysing the force equilibrium acting on the droplet interface between the fluid shear force (*F_S_*) and Laplace pressure (*F_L_*). To obtain the fluid shear force and Laplace pressure, the droplet diameter should be determined. Assuming that the droplet geometry is spherocylinder, the droplet diameter can be obtained by solving Equation (1):

(1) $$D=\sqrt{\frac{4ab}{\pi }-a^{2}\left(\frac{4}{\pi }-1\right)}$$

where *a* and *b* are the width and length of the spherocylinder, as shown in Figure 11.

To obtain the droplet diameter (*D*), the width of the droplet (*a*) is assumed to be the same as the channel width, which is 90 µm. Meanwhile, the length of the droplet (*b*) is assumed to be the average droplet length obtained in the current setup, which is 112.95 µm. Putting both variables into Equation (1) results in the droplet diameter (*D*) = 103.74 µm.

Afterwards, the shear force and Laplace pressure can be obtained from Equations (2) and (3), respectively:

(2) $$F_{s}=\mu \times u$$

(3) $$P_{L}=\frac{2\gamma }{R}$$

where the dynamic viscosity (μ) of the carrier phase is 0.03 Pa.s and the velocity (*u*) of the carrier phase is 0.036 m/s. Putting those variables into Equation (2) results in 0.0011 N of shear force ($F_{s}$). 

On the other hand, to obtain the Laplace pressure, the surface tension (γ) is 0.03 N/m. Putting all variables into Equation (3) results in a Laplace pressure ${(P}_{L})$ of 388.86 Pa. 

The Laplace pressure is then multiplied by the droplet surface area to obtain the equivalent force by putting those variables into Equation (4), which results in 0.000029 N.

(4) $$F_{L}=P_{L}\times A=P_{L}\times \pi \times \frac{D^{2}}{4}$$

Retrieving this data, we have proven that $F_{L}<F_{S}$, which means that the droplets are indeed stable and would not break due to outside forces, thereby proving that the droplets are viable.

The experimental results show that an increase in the Reynolds number of the continuous phase leads to a decrease in the size of the droplets and an increase in the Reynolds number of the dispersed phase leads to an increase in the size of the droplets. These results are supported by a computational and experimental study on the flow rate effect on droplet generation in a flow-focusing geometry [36,37]. Their findings suggest that at a constant flow rate ratio between the continuous and dispersed phases, a higher dispersed phase flow rate decreases the droplet diameter and spacing. Additionally, at a constant dispersed phase flow rate, a higher continuous phase flow rate decreases the droplet diameter.

In the current study, polycarbonate and polydymethylsiloxane droplet generator chips are used in serial to produce double layer droplet emulsification. Droplet generation can also be characterized by capillary number, where at Ca > 0, droplets were formed in the dripping regime. The second droplet emulsification also managed to encapsulate multiple droplets in one go, due to the large size difference in the channels of the first and second chips. The output of our system realized droplets with sizes of 500–1000 µm, whereas the expected criteria to be applied in stem cell application were around 100–500 µm [38]. This two-stage approach eliminates the need to selectively treat the microchannel to facilitate flow of both aqueous and organic phases compared to the single stage double droplet generator chip [39].

Experimental works on double emulsion droplets demonstrated that a higher flow rate of the carrier phase decreased the size of the external double emulsion droplets [40,41]. The formation of double emulsion droplets using a series of two separated PDMS microfluidic devices was reported [42]. Such an approach allows for different surface treatments for each device to optimize droplet formation. The utilization of a commercial droplet generator chip (ChipShop GmbH, Jena, Germany) to generate double emulsion droplets was reported, where single emulsion droplets were formed using the commercial chip followed by the formation of double emulsion droplets using vortex mixing [43]. The fabrication of double emulsion droplets for food/pharmaceutical applications using a glass-capillary device was reported [44]. Table 4 shows a comparison of the flow rate and drop sizes of a double-emulsion-droplets system from the current study and other studies. The current study demonstrates the formation of double emulsion droplets with a relatively low flow rate compared to other studies [40,41,42,43,44,45]. The inner single emulsion drop diameter of the current study is comparable to that of other studies. The phenomenon of spheroidal droplets produced from the plug form as shown in Figure 10 also confirmed the results of a previous study by Saeki [46]. Meanwhile, the outer double emulsion drop diameter in the current study is relatively larger compared to other studies, which makes the results of the current study applicable for bioink production in bioprinting technology, where bulk encapsulation of cells is preferred [47]. Comparison of droplet size from previous studies and current result is presented in Table 5. This application shall also be beneficial for drug delivery research and organoids testing. 

## 5. Conclusions

The two-step double-emulsification system shows promise, with the current setting managing to encapsulate multiple droplets in one go. By using the two-step double-emulsification system, the flow rates of each process can be tuned independently, offering a wider range of droplet sizes that can be produced. The current final droplet sizes available range from 500–1000 µm. The sizes are tunable by controlling the flowrate ratio of the disperse or continuous mediums to be between around 0.5–2 mL/h. A higher Reynolds number and capillary number will increase the droplet size, which is indicated by plug length over width of channel. The existing commercial polycarbonate chip is suitable for the first stage of emulsification due to the hydrophobic behavior of the substrate and the aqueous medium. Moreover, the flexibility of the surface treatment has made PDMS an ideal option for the second stage of emulsification, with a relatively easy surface treatment. In the meantime, the viability of living cells inside the alginate-oil-water mixture is being investigated. A positive result will enable further experiments to deploy these cells in a future experimental direction.

## Figures and Tables

**Table 1:** Properties of fluid phases.

Materials	Density (kg/m^3^)	Viscosity (kg/ms)
Alginate	1600	0.02
Water	998.2	0.001003
Mineral Oil	840	0.014

**Table 2:** Parameter variation for stage 1 emulsification.

Flow Rate [mL/h]		Reynolds Number [Re]		Capillary Number [Ca]	
Oil	Water	Oil	Water	Oil	Water
0.2	0.1	0.07	0.60	0.00899	0.00032
0.2	0.2	0.07	1.19	0.00899	0.00064
0.2	0.3	0.07	1.79	0.00899	0.00096
0.2	0.4	0.07	2.38	0.00899	0.00129
0.1	0.2	0.04	1.19	0.00449	0.00064
0.2	0.2	0.07	1.19	0.00899	0.00064
0.3	0.2	0.11	1.19	0.01349	0.00064
0.4	0.2	0.14	1.19	0.01798	0.00064

**Table 3:** Parameter variation stage 2 emulsification.

Flow Rate [mL/h]		Reynolds Number [Re]		Capillary’s Number [Ca]	
Oil	Water	Oil	Water	Oil	Water
1.4	0.7	0.07	0.60	0.00124	0.00004
1.4	1.4	0.07	1.19	0.00124	0.00009
1.4	2.1	0.07	1.79	0.00124	0.00013
1.4	2.8	0.07	2.38	0.00124	0.00018
0.7	1.4	0.04	1.19	0.00062	0.00009
1.4	1.4	0.07	1.19	0.00124	0.00009
2.1	1.4	0.11	1.19	0.00185	0.00009
2.8	1.4	0.14	1.19	0.00247	0.00009

**Table 4:** Parameter variation at stage 2 emulsification.

Re of Alginate	Re of Oil	Re of Water	Generation Mode	Measured Alginate Droplet Diameter (Microns)	Calculated Alginate Droplet Diameter (Microns)
1.19	0.04	1.19	N/A	N/A	N/A
1.19	0.07	1.19	Squeezing	112.95	103.74
1.19	0.07	1.5	Squeezing	49.01	58.58

**Table 5:** Comparison of flow rate and drop size of double emulsion droplets production.

Inner Droplet Flow Rate (mL/h)	Outer Droplet Flow Rate (mL/h)	Inner/Core Droplet Diameter (µm)	Outer Droplet Diameter (µm)	Ref.
0.05–0.25	0.02–0.1	~40	~126–159	[39]
N/A	~5–15 × 10^3^	~52	~83	[40]
0.18	0.6	~45	~59	[42]
~1000	~3–12 × 10^3^	N/A	~100–180	[43]
0.3–0.8	0.6–1.6	~65–90	~150	[44]
~0.1–0.5	~0.7–2.4	~50–100	~500–1000	Current study

## Data Availability

Not applicable.

## Supplementary Materials

The following supporting information can be downloaded at https://www.mdpi.com/article/10.3390/mi14101828/s1.
